# The efficacy of hyaluronic acid treatment on induced periodontitis in rats exposed to gamma radiation

**DOI:** 10.1038/s41598-024-82239-4

**Published:** 2025-04-10

**Authors:** Salwa Farid Ahmed, Amira Ibrahim Sayed, Heba Abdelfatah, Lobna Mohamed Abdel-Aziz

**Affiliations:** 1 Health Radiation Research Department, National Centre for Radiation Research and Technology, Egyptian Atomic Energy Authority, Cairo, Egypt; 2 Oral and Dental Biology Department, Faculty of Dental Medicine for Girls, Al-Azhar University, Cairo, Egypt; 3 Oral Medicine, Periodontology, Oral Diagnosis and Radiology Department, Faculty of Dental Medicine for Girls, Al-Azhar University, Cairo, Egypt

**Keywords:** Gamma radiation, Periodontitis, Hyaluronic acid, Bone density, CBCT, Diseases, Health care

## Abstract

The periodontium is one of the radiation-sensitive tissues; the periodontal membrane’s vascularity and cellularity were reduced, and the danger of losing periodontal attachment was raised. This study was performed to evaluate the efficacy of hyaluronic acid treatment on induced periodontitis in rats exposed to gamma radiation radiographically and histopathologically. A total number of 30 adult male Albino rats were divided randomly into five groups (n = 6). Group 1 (C): received neither irradiation nor treatment. Group 2 (P): was subjected to induced periodontitis. Group 3 (PT): subjected to induced periodontitis with hyaluronic acid treatment. Group 4 (RP): received a single dose of total cranium irradiation 20 Gy with induced periodontitis. Group 5 (RPT): received a single dose of total cranium irradiation 20 Gy with induced periodontitis and hyaluronic acid treatment. All animals were euthanized, and the outcomes of treatment were evaluated radiographically by cone beam computed tomography (CBCT) and histopathologically. Results: Comparison of the five groups about bone density by one-way ANOVA showed a significant difference among groups (P < 0.001). The highest bone density values were measured in Group (PT) (1245 ± 22.86), while the lowest bone density values were measured in Group (RP) (926 ± 31.47). Using post hoc analysis for pairwise comparisons showed that Group (PT) and Group (RPT) have significantly higher values than Group (P) and Group (RP) (P < 0.001). Histologically, the group (RPT) shows a new formation of irregular connective tissue fibers of the periodontal ligament (PDL) with an area of distortion, fibrous marrow spaces with wide osteocyte lacunae without nuclei, and Haversian canals with empty blood vessels. The radiographic and histopathological findings of using HA as a topical application in rats subjected to induced periodontitis and exposed to gamma radiation revealed enhanced healing ability of the periodontal tissue with restoration of the bone density. Depending on these results, HA could be used as an adjunct local delivery agent for periodontal-affected patients receiving radiotherapy.

## Introduction

One of the major challenges facing medicine in the twenty-first century is the increasing incidence and diversity of cancer development. Without a doubt, the treatment protocols for this illness have advanced significantly. These protocols currently make up immunotherapy, hormone therapy, chemotherapy, radiotherapy, surgery, and/or chemotherapy in combination. Radiotherapy is one of the most effective cancer treatment options among those already addressed^1^.

One radiation-sensitive tissue is periodontium. Increased risk of periodontal attachment loss and decreased blood supply and cellularity of the periodontal membrane are the direct and indirect effects of high-dose radiation on the periodontium. Furthermore, endarteritis and hypovascularity in the alveolar bone enhance the possibility of osteoradionecrosis development, particularly in those with periodontitis^2^. The symptoms are all attributed to reactive oxygen species (ROS), which damage periodontal tissue by triggering the expression of nuclear factor-kappa b, releasing pro-inflammatory cytokines, and activating the receptor activator of nuclear factor kappa beta ligand^3^.

Periodontitis is one of the factors that affect implant survival, whether it occurs before or after implant insertion. Proper periodontal management, including professional and home maintenance, could enhance the survival of the implant in terms of the absence of peri-implant mucositis and peri-implantitis, thus improving the patient’s life quality^4^. The key element in achieving long-term periodontal outcomes is the elimination or reduction of periodontal pathogens such as Aggregatibacter actinomycetemcomitans and Porphyromonas gingivalis. Full-mouth subgingival instrumentation could effectively reduce such periodontal pathogens that are confirmed clinically with a significant reduction of the periodontal pocket depth^5^.

The chronic biofilm-associated bacterial infections of periodontal tissues that cause periodontitis are what cause the immune system to release pro-inflammatory cytokines such as interleukin-1 and tumor necrosis factor-α. This results in a marked increase in the number and activity of polymorph nuclear cells, which release ROS and proteinases^6^.

Natural non-sulfate glycosaminoglycans, or hyaluronic acid (HA), are linear polysaccharides derived from the extracellular matrix of several bodily tissues and organs, including connective tissue, synovial fluid, and embryonic mesenchymal cells. Excellent biophysical properties of HA include its high viscosity, elasticity, and hygroscopicity. Additionally, HA plays a part in various essential biological processes, such as signal transduction, proliferation, and differentiation. HA is an integral part of the extracellular matrix and is necessary for many biological functions, including the healing of wounds^7^.

HA promotes tissue healing by stimulating and reducing inflammatory processes and enhancing cell migration, proliferation, and angiogenesis^8^. HA acid plays a role in controlling the inflammatory process; this action depends on its molecular weight. High molecular weight (HMW) HA at the inflammation stage is aggressively decomposed into oligomers of low molecular weight (LMW) HA, which in turn promotes leukocyte chemotaxis and expression of inflammatory cytokines like IL-1β, TNF-α, and IGF-1. HMW HA displays anti-angiogenic and anti-inflammatory properties, whereas LMW HA acts oppositely, being pro-inflammatory and pro-angiogenic^9^.

Jokela et al.^10^ demonstrated that the stimulation of keratinocytes with inflammatory molecules, such as TNF-α, IL-1, or high glucose concentration, induced the formation of HA cables and the adhesion of leukocytes without alteration in the secretion of HA. Such a rearrangement of HA structure may have a protective function during inflammation; monocytes can bind HA cables regardless of their activation state, avoiding contact with inflammation-promoting receptors^9^.

HA removes prostaglandins, metalloproteinases, and other bioactive compounds, as it has scavenging effects that lead to inflammation reduction^11^. In contrast to different growth factors used for bone healing, HA is beneficial due to its low immunogenicity and good biocompatibility^12^.

Previous studies demonstrated the use of HA to treat periodontitis due to its unique biological implications. The current study aimed to evaluate the efficacy of hyaluronic acid treatment on induced periodontitis in rats exposed to gamma radiation radiographically and histologically.

## Methods

### Ethical consideration

In this randomized controlled animal model trial, all animals were investigated following the rules and regulations of the animal experimental studies approved by the Research Ethics Committee of the National Centre for Radiation Research and Technology, Egyptian Atomic Energy Authority, Cairo, Egypt, including their facilities diet and method of scarification (57A/22).

### Sample size calculation

Based on Li et al.^12^, using the G power statistical analysis program (Version 3.1.9.4) for sample size calculation and using one-way analysis of variance (ANOVA), a total sample size of N = 30 (subdivided into 6 in each group) will be sufficient to detect a large effect size of f = 0.89 with an actual power (1 − β error) of 0.8 (80%) and primary risk of error (α = 0.05).

### Animal grouping and randomization

A total of 30 adult male Albino rats, 8–12 weeks old and weighing 200 ± 20 g, were used in this study. The animals were housed in specially designed cages (3–4 rats per cage), in a room with a 12-h day-night cycle, a temperature of 23 ± 2 °C, and humidity of 55 ± 5%. All animals were fed a semi-purified diet and water ad libitum for 10 days before the start of the experiment. The rats were divided randomly into five groups (n = 6). Group 1 (C); received neither irradiation nor treatment. Group 2 (P); was subjected to induced periodontitis. Group 3 (PT) was subjected to induced periodontitis followed by hyaluronic acid treatment. Group 4 (RP): subjected to a single dose of total cranium irradiation 20 Gy, then induced periodontitis. Group 5 (RPT): subjected to a single dose of total cranium irradiation 20 Gy, then induced periodontitis, followed by hyaluronic acid treatment.

### Radiation exposure and periodontitis induction:

The Indian Gamma Cell (60Co) was used for the radiation process at the National Centre for Radiation Research and Technology, Egyptian Atomic Energy Authority, Cairo, Egypt.

Ketamine injections (25 mg/kg) were used to anesthetize the rats in groups (P), (PT), (RP), and (RPT). The rats in groups (RP) and (RPT) were completely immobilized on a special shield, and their heads were exposed to a single dose of 20 Gy gamma radiation^13^ (dose rate: 11.07 Gy/min). A 3–0 silk suture was used to ligature the mandibular incisors’ cervical area for the mentioned 4 groups^14^. At the end of radiation, rats were returned to the Animal Care Centre of the National Centre for Radiation Research and Technology, Egyptian Atomic Energy Authority, Cairo, Egypt.

### Hyaluronic acid application

**Fig. 1:**
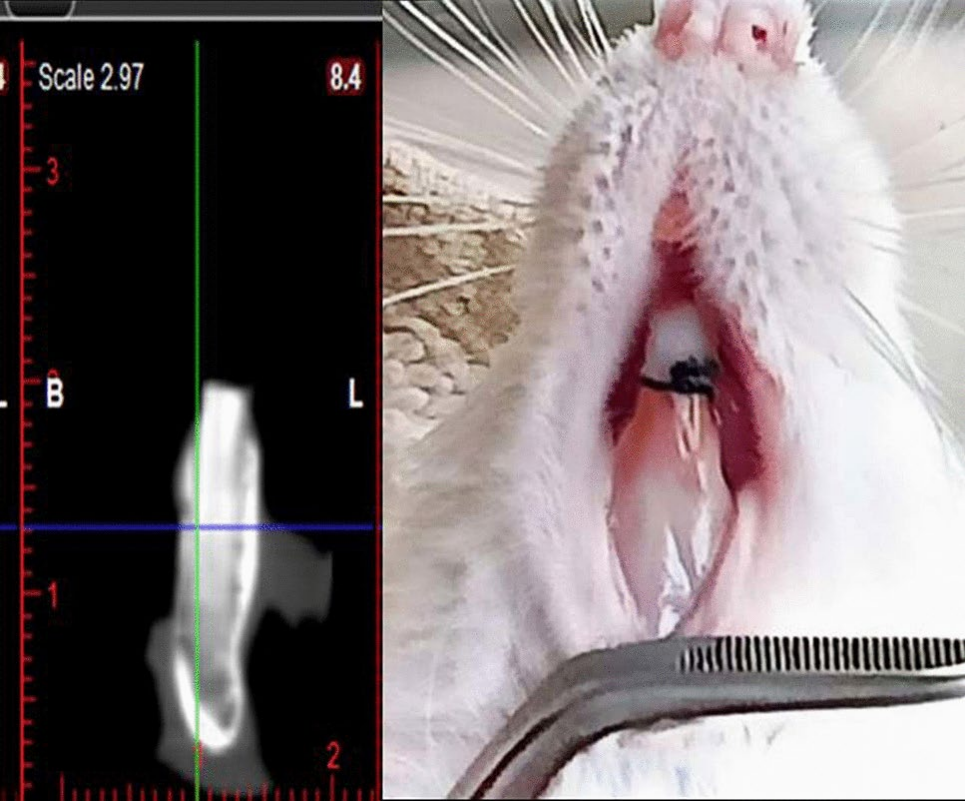
Clinical and radiographic photo of established periodontitis.

After 7 days, periodontitis was reported clinically, as there was redness and bleeding from the gingiva, and radiographically, as shown in (Fig. 1). Animals of groups (PT) and (RPT) received 0.5 ml of hyaluronic acid solution (Hyalubrix, Hyaluronic acid sodium salt 1.5% solution, Fidia Farmaceutici, Italy) topically in the sub-gingival tissues once daily for 1 week, as reported by^15^.

### Animal euthanizing

All animals of groups (P), (PT), (RP), and (RPT) were euthanized after one week of established periodontitis proven. Both groups (PT) and (RPT) were euthanized the next day after the last dose of treatment by an overdose of anesthesia (Ketamine), and the outcomes of treatment were evaluated radiographically and histopathologically.

### Radiographic analysis

A CBCT analysis was performed for each sample, and by using the Planmeca Romexis software, a calibrated and blinded examiner visualized and assessed the tomographic pictures. One mandible is kept in each of the plastic boxes holding the mandibles. With the help of an empty box, the boxes were placed inside the machine. All technical parameters were then applied and saved in Romexis software for measurements to achieve the perfect height for the tomographic scanning. Using sagittal plane axes positioned above the long axis’ center, the bone density in the periodontitis region (the space between two central incisors) was evaluated. In the density measuring method, a 0.50-cm square area was chosen so that all data could be collected and statistically examined.^16^

### Histopathological analysis

The lower jaw was separated, and then the operated anterior area, including teeth, bone, and soft tissue, was harvested and fixed in 10% buffered formalin (pH 7.4) for 24 h. Then the specimens were decalcified by EDTA and changed every 48 h for 20 days. The samples were then washed, dehydrated in ascending grades of ethyl alcohol, and finally treated by a clearing agent as xylol, followed by embedding in paraffin. Then, serial sections (5 μm) covering the entire lower incisors area from coronal to apical aspects in the buccolingual plan, throughout the mesiodistal extension of the tooth, were prepared for histological staining. Hematoxylin–eosin (H&E) was used to stain these sections^17^. In addition, an Alcian Blue (pH 2.5) stain was used for the analysis of collagen and new bone formation^18^. The Leica Qwin 500 image analyzer computer system (England) at the Pathology Department, Faculty of Dental Medicine for Girls-Al Azhar University, was used for histological examination.

### Statistical analysis

Study data were analyzed using SPSS version 26 as mean SD. P values < 0.05 were regarded as statistically significant. They were explored for normality by checking the data distribution and using Kolmogorov–Smirnov and Shapiro–Wilk tests. Values were normally distributed in all groups. Statistical analysis was done using one-way ANOVA analysis (for general comparisons) followed by post hoc pairwise comparisons.

## Results

### Radiographic analysis

Comparison of the five groups about bone density by one-way ANOVA showed a significant difference among groups (P < 0.001). The highest bone density values were measured in Group (PT) (1245 ± 22.86), while the lowest bone density values were measured in Group RP (926 ± 31.47) (Table 1).

**Table 1:** Bone density of different studied groups (n = 6).

Group	Mean	Std. deviation	95% confidence interval for mean		Minimum	Maximum	F	P value
			Lower bound	Upper bound				
C	1239	33.46	1185.74	1292.25	1211	1287	63.74	P < 0.001*
P	1076	54.68	988.99	1163.00	1017	1143		
PT	1245	22.86	1208.86	1281.63	1219	1274		
RP	926	31.47	876.16	976.33	894	958		
RPT	1229	23.30	1192.42	1266.57	1200	1251		

*Significant (P ≤ 0.05) ns; non-significant (P > 0.05).

Using post hoc analysis for pairwise comparisons showed that Group (C) has significantly higher values than Group (P) and Group (RP) (P < 0.001). Group (P) has significantly higher values than Group (RP) (P < 0.001). Group (PT) has significantly higher values than Group (P) and Group (RP) (P < 0.001). Group (RPT) has significantly higher values than Group (P) and (RP) (P < 0.001). No other significant differences were noted (p > 0.05) (Table 2).

**Table 2:** Pairwise comparisons regarding bone density using Post hoc analysis.

Group I	Group J	Mean difference (I-J)	Std. error	Sig	95% confidence interval	
					Lower bound	Upper bound
Group C	Group P	163.00*	24.83	0.000	81.39	244.60
	Group PT	− 6.25	24.83	1.000	− 87.85	75.35
	Group RP	312.75*	24.83	0.000	231.14	394.35
	Group RPT	9.50	24.83	1.000	− 72.10	91.10
Group P	Group PT	− 169.25*	24.83	0.000	− 250.85	− 87.64
	Group RP	149.75*	24.83	0.000	68.14	231.35
	Group RPT	− 153.50*	24.83	0.000	− 235.10	− 71.89
Group PT	Group RP	319.00*	24.83	0.000	237.39	400.60
	Group RPT	15.75	24.83	1.000	− 65.85	97.35
Group RP	Group RPT	− 303.25*	24.83	0.000	− 384.85	− 221.64

### Histopathological analysis

#### H&E

**Fig. 2:**
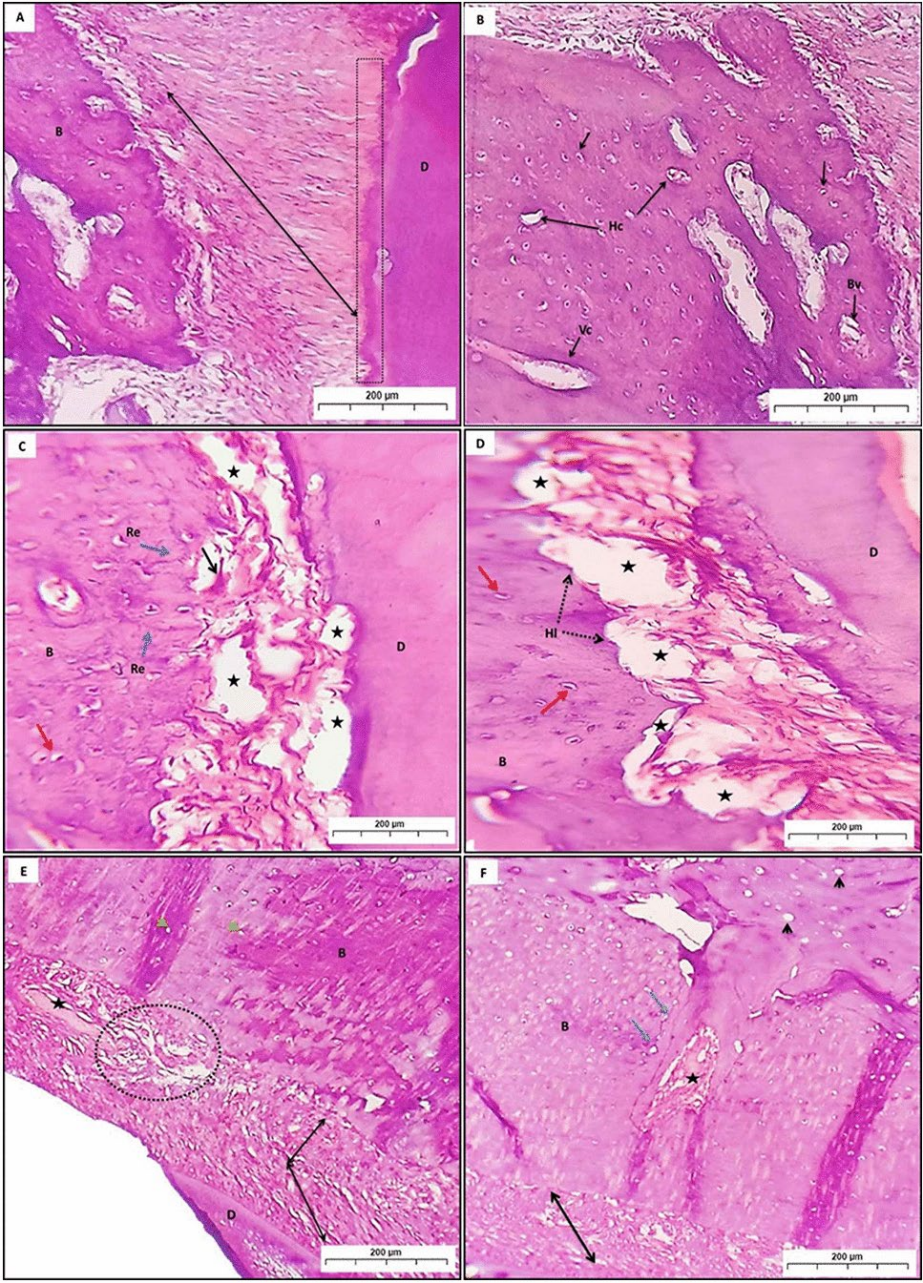
A photomicrograph (**A**) and (**B**) showing group (**C**); PDL (double head arrow). Sharpey’s fibers (dot line). Osteocytes (black arrow), Haversian canals (Hc), Volkmann’s canal (Vc), blood vessels (Bv). (**C**) and (**D**) showing group (P); loss of bone and attachment (stars). Osteoclast (black arrow). Osteocytes (red arrows), Howship’s lacunae (Hl), resting line (Re) (blue arrows). (**E**) and (**F**) showing group (PT); PDL (black arrows), new collagen formation with irregular orientation (circle). neurovascular canal (star). Empty lacunae (arrowhead), osteocytes (yellow arrows), reversal line (blue arrows). *B* bone, *D* dentin (H&E Orig. Mag. × 100).

In group (C), the periodontal ligament (PDL) appeared with normal thick regular CT fibers that connect the cementum to the alveolar bone. Sharpey’s fibers extend from the PDL into the acellular cementum. The normal appearance of compact bone holds many osteocytes with normal lacunae, many Haversian canals, and Volkmann’s canal rich with blood vessels filled with RBCs (Fig. 2 A,B).

In group (P), loss of the bone trabeculae and PDL attachment increase the number of osteoclastic cells. Also, reversal and resting lines, as well as Howship’s lacunae, can be detected (Fig. 2C,D).

In group (PT), which was subjected to induced periodontitis with hyaluronic acid treatment, the PDL appeared thick with regular CT fibers that connect the cementum to the alveolar bone but with different directions; also, abundant new collagen synthesis with irregular orientation could be seen. Neurovascular canal appeared congested with RBCs. The bone trabeculae show empty lacunae while other sites show osteocytes; besides, the reversal line can be detected (Fig. 2E,F).

**Fig. 3:**
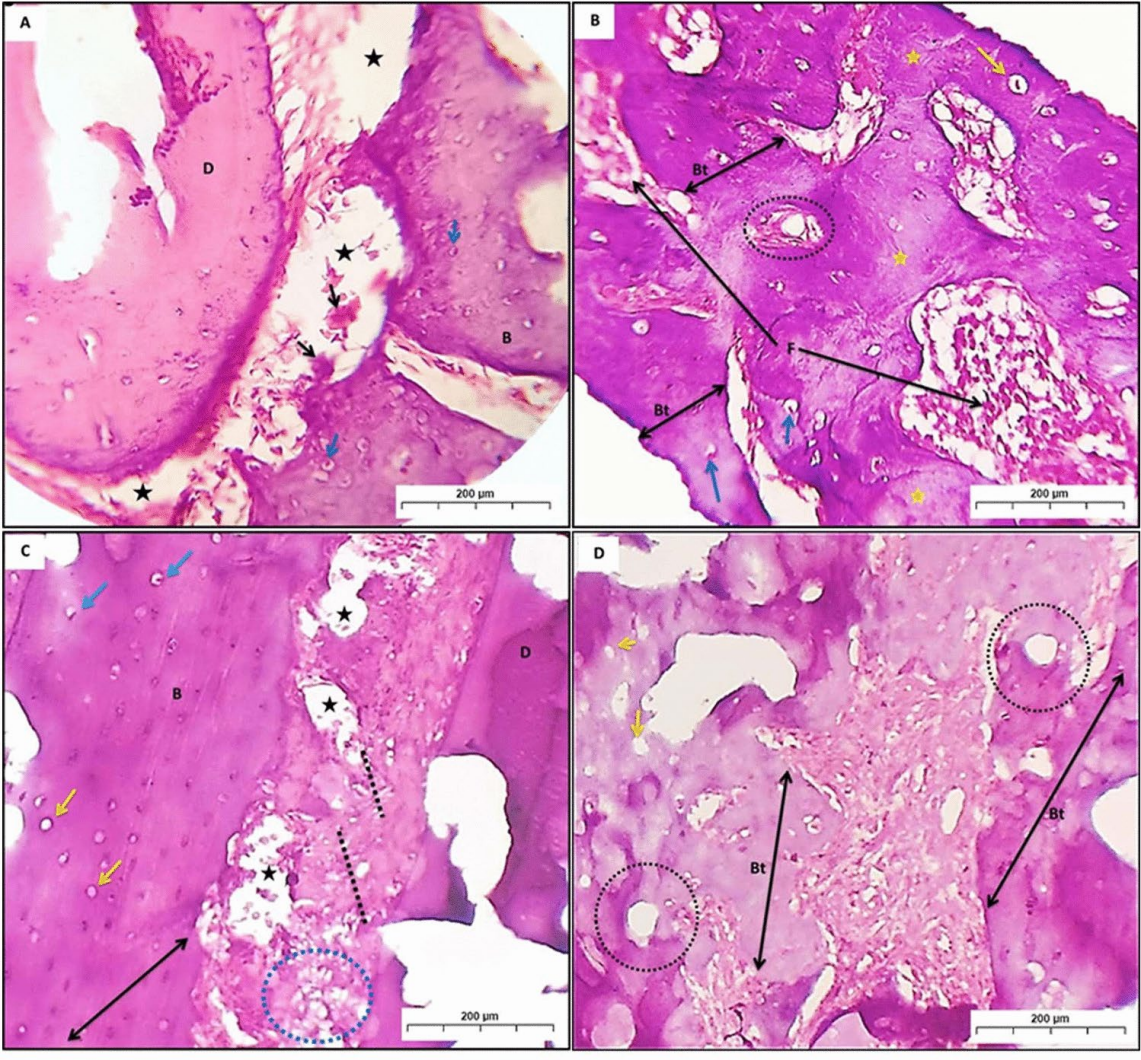
A photomicrograph (**A**) and (**B**) showed group (RP); destructed PDL (black stars). Many osteoclastic cells (black arrow). Wide osteocyte lacunae with eccentric osteocyte (blue arrow). Bone trabeculae (Bt), entrapped osteocytes (yellow arrow), absence of osteocytes (yellow stars). Fibrous bone marrow (F). (**C**) and (**D**) revealed group (RPT) with irregular connective tissue fibers of PDL (dot lines) with area of distortion (blue circle). (**C**) Necrotic bone (double head arrows), areas of periodontal tissue degradation (black star). Wide osteocyte lacunae with empty nuclei (yellow arrows). Empty Haversian canals from blood vessels (black dot circle) (**D**), *B* bone, *D* dentin. (H&E Orig. Mag. × 100).

Group (RP) showed loss of the PDL, and the bone trabeculae presented wide osteocyte lacunae with eccentric osteocytes with many osteoclastic cells. Also, another area showed a lower amount of bone trabeculae formation, lined by osteoblasts and entrapped osteocytes, whereas some areas showed a complete absence of osteocytes. The haversian canal can be noticed with fibrous bone marrow space (Fig. 3A,B).

In group (RPT), the PDL appeared irregular with different orientations of connective tissue fiber, while some areas showed distortion of the PDL. Fragments of necrotic bone with wide osteocyte lacunae, empty nuclei, and areas of periodontal tissue degradation can be noticed. Some areas showed wide osteocyte lacunae with empty nuclei. Haversian canals appeared empty of blood vessels (Fig. 3C,D).

#### Alcian blue (pH 2.5)

Alcian blue is a stain used to identify acidic substances within tissues. It stains glycosaminoglycans (GAGs) and mucopolysaccharides, including hyaluronic acid, a pale pink color. Meanwhile, it stains nuclei blue. Tissue regeneration is the process by which damaged or lost tissue is replaced with new tissue. When this process is enhanced, the thickness of the regenerated tissue is increased. As more cells are generated, the tissue becomes thicker, and over time the Alcian blue stain becomes deeper.

**Fig. 4:**
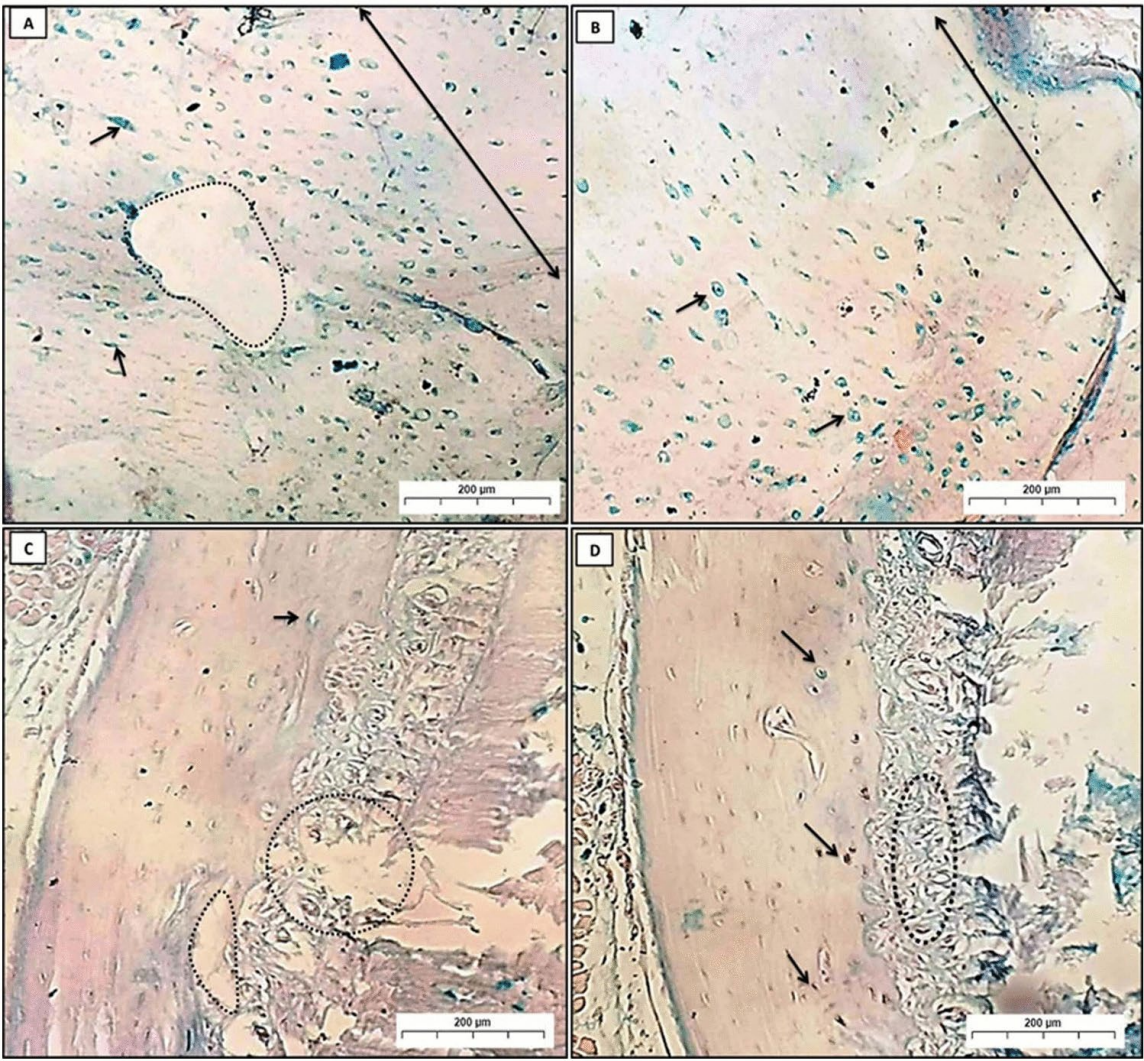
A Photomicrographs (**A**) and (**B**); showed group (**C**); homogenous compact bone (double-headed arrow), osteocytes (arrow). (**C**) revealed group (P); osteocytes (arrow), destruction in bone, and PDL (dot circles). (**D**) Represented group (PT); osteocytes (arrow), restoring of PDL (dot circles) (Alcian blue Orig. Mag. × 100).

In group (C), a positive Alcian blue reaction was noticed as blue stains in all osteocytes, with high distribution all over the bone trabeculae (Fig. 4A,B). In group (P), a positive Alcian blue reaction was seen as blue deposits in some osteocytes and osteoblasts, which were sparsely dispersed in the small amount of bone (Fig. 4C). Group (PT) showed variable degrees of stainability ranging from strong to moderate positive reactions in the newly formed bone trabeculae (Fig. 4D).

**Fig. 5:**
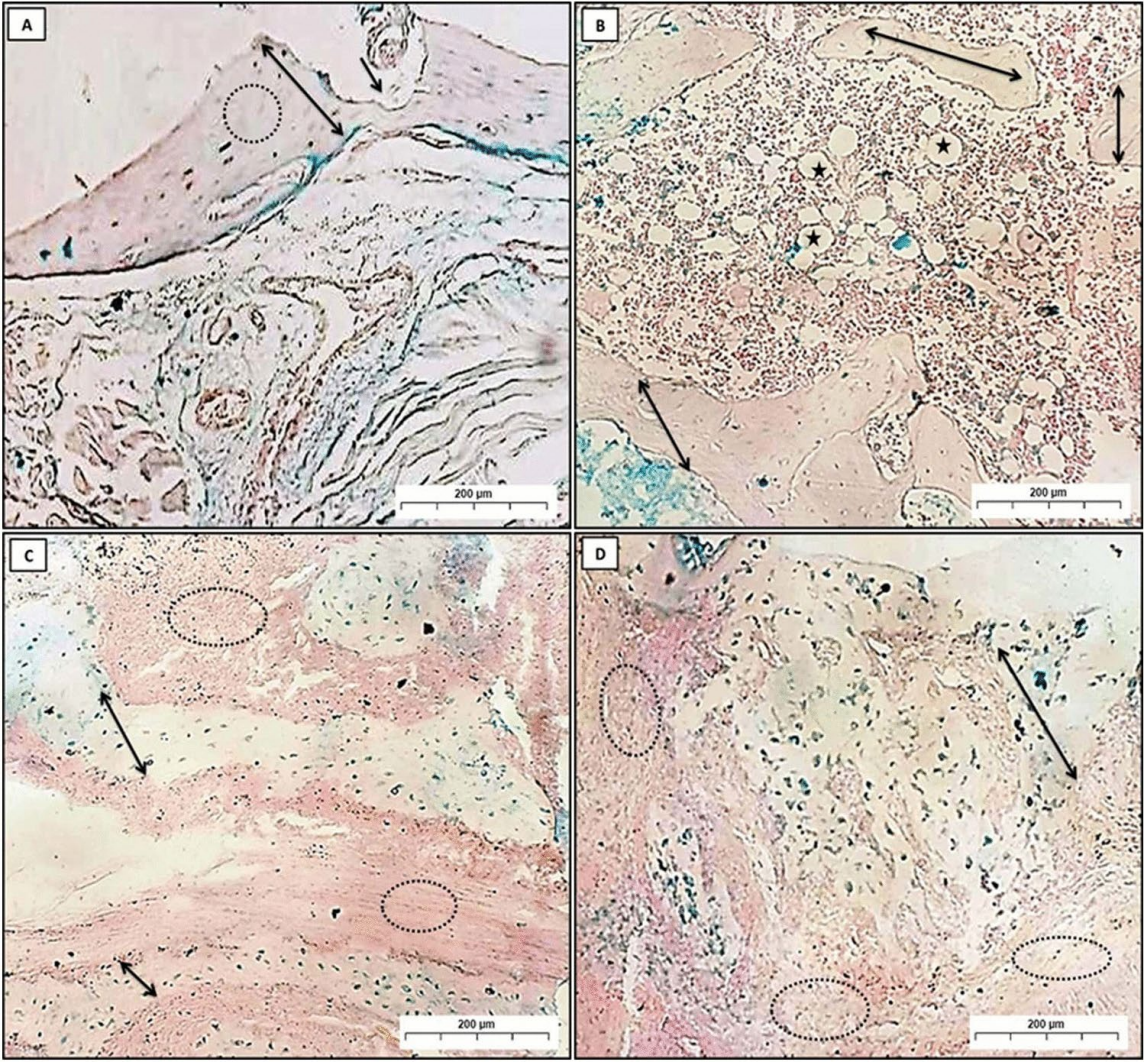
Photomicrographs (**A**) and (**B**) showed group (RP); In photomicrograph (**A**), compact bone (double-headed arrow), area of bone destruction (black arrow), and absence of osteocytes (dot circles). Photomicrograph (**B**) showed a thin small bone trabecula of spongy bone (double-headed arrow) and fatty bone marrow (stars). In groups (RPT) (**C**) and (**D**); newly formed bony spicules (double-headed arrow), with many collagens’ fibers formation (dot circles) (Alcian blue Orig. Mag. × 100).

In group (RP), thin layers of compact bone appeared destroyed, and the blue deposits were diminished all over the bone area (Fig. 5A,B). Whereas in the group (RPT), thin layers of newly formed bone trabeculae of spongy bone showed positively stained areas with Alcian blue. Moreover, some areas of newly formed bone had empty lacunae, which show negative reactions (Fig. 5C,D).

## Discussion

To our knowledge, there are no studies on the effect of topical hyaluronic acid application as a treatment for periodontitis in gamma-irradiated rats. The challenge lies in confronting two factors that negatively affect the periodontal ligament and alveolar bone, namely periodontitis and exposure to gamma radiation, which is common in head and neck cancer patients. The most significant findings of this study include the effectiveness of hyaluronic acid in treating induced periodontitis in gamma-irradiated rats by restoring alveolar bone density, improving the periodontium’s histological structure, and promoting the growth of new bone.

The prevention of periodontitis or peri-implant disease is much better than treatment in the case of diabetes, radiotherapy, or dental implant placement. Preventive measures aim to prevent the establishment of risk factors rather than addressing them after they have developed. Such measures include glycemic control, cessation of cigarette smoking, improved oral hygiene, regular supportive periodontal care, and reducing bruxing and parafunctional habits. However, the occurrence of periodontitis, peri-implant mucositis, or peri-implantitis is inevitable in many cases and requires effective treatment. There are many treatment methods, including non-surgical mechanical/physical therapy in addition to antibiotics, topical antiseptics, chemical agents, or photodynamic therapies in case of peri-implant mucositis. As for peri-implantitis, non-surgical submarginal instrumentation is a suitable choice. It could be mechanical using instruments, lasers, or air-polishing. Also, it could be chemical using antimicrobial photodynamic therapy or antiseptic desiccant solution. Such instrumentation can be augmented with the use of adjunctive locally administered antimicrobials, systematically administered antibiotics, or probiotics^19^.

The ligature-induced periodontitis used in that study serves as a retentive factor for bacterial plaque, which leads to the early development of periodontitis^20^. Special skill is needed to apply this method on rats` molars as inserting the ligature into the interproximal spaces can be challenging due to the small size of the tooth, limitation in viewing the sulcus region, and the tiny oral space in which to perform knotting and insertion^21^. Hence, we conducted this study on rats’ incisors.

In addition to the verified outcomes, this approach offers additional benefits such as less invasive surgery, less operating trauma, easy ligature integrity monitoring, and daily tissue changes without additional trauma or general anesthesia^22^.

Radiotherapy is documented as a palliative measure for cancers that are incurable in their late stages or as an adjuvant with chemotherapy after tumor excision^23^. Radiation therapy to the head and neck region causes dry mouth and changes the oral microbiota, which makes periodontitis more likely to arise. Sometimes tooth extraction is the only solution available for aggravating periodontitis after radiotherapy, which may lead to osteoradionecrosis^24^. The high dosage fraction of radiation has a localized effect on the periodontium, changing its cellularity and vascularity and reducing its ability to repair^25^.

Hyaluronic acid is a naturally occurring linear polysaccharide found in all periodontal tissues in varying quantities. It has a structural role as a part of tissue architecture and a modulating role as a regulator of cell transduction, differentiation, adhesion, and proliferation^26^.

The bone density showed a significant reduction in the (P) group with a more significant reduction in the (RP) group. These results followed Toraman et al.^27^, and Araújo et al.^28^, who reported significant bone loss in the ligature-induced periodontitis group compared to the control. Mostafa et al.^29^ found that periodontitis followed by gamma irradiation exposure induced more significant bone density reduction as compared to periodontitis alone. However, by comparing the bone density of the (C) group to the (PT) and (PRT) groups, there was no significant difference among the three groups. This result agreed with Sarkarat et al.^30^, who reported that the proper effect of HA on bone regeneration is visible at an early phase of healing because HA is an element of the extracellular matrix that acts as a scaffold for mesenchymal cell migration, allowing them to differentiate, proliferate, and migrate, which in turn induces the growth of osteoblasts and osteocytes.

In our study, both groups (P) and (RP) showed loss of the bone trabeculae and PDL attachment, many osteoclastic cells, reversal, and resting lines. Besides, areas with lower amounts of bone trabeculae, some bone trabeculae showed wide osteocytic lacunae with eccentric osteocytes, areas with a complete absence of osteocytes, and haversian canals with fibrous bone marrow space were detected. These results agreed with Salem et al.^31^, and Abu El-Azayam et al.^32^ studied the effect of ligature on periodontal tissue. As well, these results agree with Ahmed^33^, who studied the effect of a low dose of gamma irradiation on PDL. He found that the PDL shows vacuolization and decreased osteocyte number, fibroblast cell, and collagen area.

We found that topical HA treatment in (PT) and (RPT) groups revealed restoration of PDL and alveolar bone. This effect was interpreted by Fallacara et al.^34^ and Vigetti et al.^35^, as HA improves the healing of induced periodontitis through two simultaneous mechanisms, passive and active. The passive one is related to its anti-inflammatory potential that augments the healing process, while the active one is mediated via modulation of signal transduction provoked through the binding of the ligand and its receptors, releasing growth factors responsible for cell proliferation, differentiation, migration, and extracellular matrix protein synthesis.

HA plays a significant role in the early phase of the healing process, including stimulation of the release of pro-inflammatory cytokines, the release of fibroblasts from the extracellular matrix, and an increase in the migration of cells^36^. Moreover, HA has a high capacity to increase bone morphogenetic protein-2 activity, a member of the TGF-β superfamily, which plays a role in enhancing the differentiation of mesenchymal cells into osteoblasts^37,38^. In addition to these effects, the application of HA as an adjunct to basic periodontal therapy significantly increases the total antioxidant capacity, enabling the counteracting of free radicals generated by gamma irradiation^39^.

Our results about the beneficial effect of HA on periodontitis followed many studies. Mesa^40^ found that HA gel treatment of patients with chronic periodontitis induced a significant reduction in the proliferation index of the gingival epithelium, including the fibroblasts and lymphocytes, which led to a receding in existing inflammation and diminishing gingival bleeding index with an overall improvement in the periodontitis.

Ibraheem et al.^41^ said that subgingival application of 0.8% HA gel with basic scaling and root planning significantly decreased the bleeding on probing and inhibited bacterial recolonization, which augments its use as adjuvant therapy for chronic periodontitis. Moreover, Gangadhar et al.^42^ proved a significant improvement in the clinical parameters recorded, including gingival index, plaque index, probing pocket depth, and clinical attachment level.

Hence, the results indicate the ability of topical hyaluronic acid to treat induced periodontitis in rats exposed to gamma radiation, as it restores bone density while improving the tissue structure and stimulating the construction of new bones, which makes it a suitable option in cancer patients who are exposed to radiotherapy as part of the treatment protocol.

## Conclusions

The radiographic and histopathological findings of using HA as a topical application in rats subjected to induced periodontitis and exposed to gamma radiation revealed enhanced healing ability of the periodontal tissue with restoration of the bone density. Depending on these results, HA could be used as an adjunct local delivery agent for periodontal-affected patients receiving radiotherapy.

## Data Availability

All data generated or analyzed during this study are included in this published article".
